# Impact of irrigation water quality on human norovirus surrogate survival during leafy green production

**DOI:** 10.3389/fpls.2023.1128579

**Published:** 2023-04-03

**Authors:** Xi Wu, Anne-laure Moyne, Thais De Melo Ramos, Linda J. Harris, Erin DiCaprio

**Affiliations:** ^1^ Department of Food Science and Technology, University of California, Davis, Davis, CA, United States; ^2^ Western Center for Food Safety, University of California, Davis, Davis, CA, United States

**Keywords:** norovirus, produce, irrigation water, soil, tulane virus (TV), murine norovirus (MNV), foodborne virus

## Abstract

**Introduction:**

The impact of water quality on the survival of human norovirus (NoV) was determined in irrigation water field run-off (tail water) and well water from a representative Central Coast vegetable production site in the Salinas Valley, California.

**Methods:**

Tail water, well water, and ultrapure water samples were inoculated separately with two surrogate viruses for human NoV—Tulane virus (TV) and murine norovirus (MNV)—to achieve a titer of 1×105 plaque forming units (PFU)/ml. Samples were stored at 11, 19, and 24°C for 28 days. Additionally, inoculated water was applied to soil collected from a vegetable production site in the Salinas Valley or to the surface of growing romaine lettuce leaves, and virus infectivity was evaluated for 28 days in a growth chamber.

**Results:**

Virus survival was similar for water stored at 11, 19, and 24°C and there was no difference in infectivity based on water quality. After 28 days, a maximum 1.5 log reduction was observed for both TV and MNV. TV decreased by 1.97-2.26 log and MNV decreased by 1.28- 1.48 logs after 28 days in soil; infectivity was not influenced by water type. Infectious TV and MNV were recovered from lettuce surfaces for up to 7 and 10 days after inoculation, respectively. Across the experiments there was no significant impact of water quality on the stability of the human NoV surrogates.

**Discussion:**

Overall, the human NoV surrogates were highly stable in water with a less than 1.5 log reduction over 28 days and no difference observed based on the water quality. In soil, the titer of TV declined by approximately 2 logs over 28 days, while MNV declined by 1 log during the same time interval, suggesting surrogate-specific inactivation dynamics in the soil tested in this study. A 5-log reduction in MNV (day 10 post inoculation) and TV (day 14 post inoculation) was observed on lettuce leaves, and the inactivation kinetics were not significantly impacted by the quality of water used. These results suggest that human NoV would be highly stable in water, and the quality of the water (e.g., nutrient content, salinity, and turbidity) does not significantly impact viral infectivity.

## 1 Introduction

Human norovirus (NoV) is a major cause of foodborne illness in the United States, estimated to contribute to over 58% of foodborne illnesses and resulting in an estimated five million food-related illnesses each year (Scallan et al., 2011). Human NoV causes acute gastroenteritis, with symptoms including nausea, vomiting, abdominal cramps, and diarrhea that typically last 1 to 3 days. Human NoV is transmitted by the fecal-oral route, and secondary transmission of the virus by contaminated food and water is common (Hirneisen and Kniel, 2013a; DiCaprio et al., 2015a). Human NoV is a member of the viral family *Caliciviridae* and all viruses within this family are non-enveloped and have a single-stranded, positive-sense RNA genome (Koopmans et al., 2002; Vasickova and Kovarcik, 2013; Kang, 2017; Graziano et al., 2019). Generally, non-enveloped viruses are more stable outside of the host compared with enveloped viruses that have a host-cell derived lipid bilayer coating the viral particle (Graziano et al., 2019). Human NoV has been shown to be stable for weeks on environmental surfaces and for months or longer in aqueous mediums (Zhu et al., 2020). The environmental stability of human NoV, coupled with the prevalence of asymptomatic infections and low median infectious dose, contribute to the high incidence of foodborne infections (Barclay et al., 2014).

High-risk foods for human NoV contamination include shellfish, ready-to-eat foods, and produce (DiCaprio et al., 2012). A majority of shellfish outbreaks are traced to human NoV contamination during production where human sewage pollutes growing waters (Centers for Disease Control and Prevention, 2021). In contrast, for human NoV outbreaks associated with ready-to-eat foods, the contamination occurs during preparation (Daniels et al., 2000). In these outbreaks, infected food handlers cross contaminate foods or food contact surfaces. The most frequent point of contamination is less clear for human NoV outbreaks associated with produce. Many outbreaks are traced to contamination that occurs *via* food handlers during final preparation (Grove et al., 2015). Outbreaks are infrequently traced to human NoV contamination during produce production (Callejón et al., 2015; Marsh et al., 2018). However, a high number of human NoV outbreaks associated with produce are unable to be traced to a particular infected food-handler or other contamination source (Hardstaff et al., 2018). There is a possibility that human NoV contamination during production contributes to these uncharacterized outbreaks. Since the virus remains infectious for long periods outside of the body, should contamination occur in the field, it is feasible that infectious virus could be present in produce to the time of consumption.

Water is utilized for several purposes in the produce preharvest environment, most notably irrigation. Water is a frequent source of pathogens that subsequently contaminate produce, and the Food Safety Modernization Act (FSMA) Produce Safety Rule (PSR) requires monitoring of agricultural water supplies to meet certain microbial standards (Federal Register, 2015). The preharvest agricultural water requirements under FSMA are currently under revision. However, continued testing of preharvest agriculture water for generic *Escherichia coli* or other indicators of microbial quality is likely to continue for growers in some capacity. There is poor correlation between the detection of fecal indicator bacteria and enteric viruses. For example, human NoV has been detected in well and surface water sources meeting U.S. Environmental Protection Agency (EPA) drinking water quality standards (Borchardt et al., 2003; Okoh et al., 2010).

California is the leading U.S. producer of several types of fresh market produce, with the Central Coast termed the “nation’s salad bowl” due to the high production of leafy greens in this region (Fuller, 2016). California has experienced severe droughts spanning 2013 to 2016 and again beginning in 2020, hence water access and conservation are paramount for growers (Lund et al., 2018). Thus, there is increased interest in using alternative or recycled water sources during production. One such water source is irrigation run-off or tail water, which is excess water that runs off a field after overhead sprinkler, furrow, or flood irrigation and is often collected in holding ponds. This tail water transports nutrients, sediments, and pesticides, which degrades the quality of downstream creeks, rivers, and coastal estuaries, and therefore it cannot be discharged into local watersheds. Reusing tail water in agricultural production can minimize offsite impacts of irrigation run-off while conserving surface and ground water sources. There is interest in using tail water for pre-irrigation or germination; however, due to a lack of understanding of the ability of pathogens to survive in tail water, most growers are hesitant to use tail water for irrigation of food crops.

As human NoV is difficult to cultivate *in vitro*, murine norovirus (MNV) and Tulane virus (TV) are commonly used as surrogates. MNV is a member of the genus *Norovirus* and TV is a member of the genus *Recovirus*, both in the family *Caliciviridae* (DiCaprio et al., 2012; Hirneisen and Kniel, 2013a). Both viruses are used extensively as surrogates due their genetic and structural similarities to human NoV (Hirneisen and Kniel, 2013a; Wang et al., 2013). However, differences exist between MNV and TV; for example, sialic acid is the cellular receptor for MNV while TV attaches to type B histo-blood group antigens (HBGAs) (Hirneisen and Kniel, 2013a). Using both surrogates in the current work allows for analysis of stability variation based on virus type.

The objective of this study was to determine the influence of water quality on the infectivity of human NoV and on virus stability in water, soil, and on the surface of leafy greens (with romaine lettuce used as a model system). Tail water collected from a commercial farm was used as a “lower quality” water source with a high turbidity and organic load compared to well water sources. Tail water was compared to well water from the same location and to ultrapure water to determine the influence on human NoV stability.

## 2 Materials and methods

### 2.1 Viruses and cell culture

Tulane virus (TV) and murine norovirus-1 (MNV) were propagated in confluent monolayers of the rhesus macaque kidney cell line MK2-LLC and the murine leukemic monocyte macrophage cell line RAW 264.7 (ATCC, Manassas, VA), respectively (DiCaprio et al., 2012). MK2-LLC cells were cultured in low serum Eagle’s minimum essential medium (Opti-MEM) (Invitrogen, Carlsbad, CA), supplemented with 2% fetal bovine serum (FBS) (Invitrogen) at 37°C under a 5% CO_2_ atmosphere. Confluent monolayers of cells were cultured in T-150 flasks with a cell density of approximately 4.6 × 10^6^ cells per 150 cm^2^ flask. For growing TV stock, MK2-LLC cells in T-150 flasks were washed with Hank’s balanced salt solution (HBSS) and subsequently infected with TV at a multiplicity of infection (MOI) of 0.1. Flasks were incubated for 1 h at 37°C, and then 20 ml of Opti-MEM with 2% FBS was added. The virus was harvested 2 days post inoculation when >80% of cells were detached, and then subjected to three freeze-thaw cycles, followed by centrifugation at 1,811 ×*g* for 30 min. RAW 264.7 cells were cultured in high-glucose Dulbecco’s modified Eagle medium (DMEM) (Invitrogen) supplemented with 10% FBS, at 37°C under a 5% CO_2_ atmosphere. For growing MNV stock, confluent RAW 264.7 cells in T-150 flasks were infected with MNV at a MOI of 0.1. After 1 h of incubation at 37°C, 20 ml of DMEM with 10% FBS was added. As described above, the virus was harvested 2 days post inoculation when >80% of cells were detached, and then subjected to three freeze-thaw cycles and low-speed centrifugation at 1,811 ×*g* for 30 min.

### 2.2 Virus enumeration by plaque assay

TV and MNV were quantified by plaque assays in LLC-MK2 and RAW 264.7 cells, respectively (Lou et al., 2011; DiCaprio et al., 2012). Briefly, cells were seeded into six-well plates (Corning Life Sciences, Wilkes-Barre, PA) at a density of 2 × 10^6^ per well. After 24 h of incubation, MK2-LLC or RAW 264.7 cell monolayers were infected with 400 µl of a 10-fold dilution series of TV or MNV, respectively, and the plates were incubated for 1 h at 37°C, with gentle agitation every 10 min. The cells were overlaid with 2.5 ml of Eagle minimum essential medium (MEM) containing 5% FBS, 0.014% sodium bicarbonate, 0.5% penicillin and streptomycin, 2.5% HEPES (pH 7.7), 1% L-glutamine, 14% ultrapure water (Milli-Q Advantage A10, Millipore Sigma, Burlington, MA), and 25% agarose. After incubation for 48 h at 37°C under 5% CO_2_ atmosphere, the plates were fixed in 10% (wt/vol) formaldehyde and stained with 0.05% (wt/vol) crystal violet for visualization of viral plaques. Viral titers were expressed as mean log plaque forming unit (PFU)/ml ± standard deviation. The limit of detection for the viral plaque assay was determined to be 0.5 log PFU/ml.

### 2.3 Collection of water and soil samples from the Salinas Valley

Samples of soil, well water, and tail water were collected from one farm in the Salinas Valley. Bulk soil samples and samples of well water and tail water were collected in March 2016. Tail water was collected in sterile 1-L Nalgene bottles (Thermo Scientific, Rochester, NY) attached to a telescoping pole to reach the water, as described in EPA method 1603 (US Environmental Protection Agency, 2014). An automated peristaltic pump was used to sample well water from the sprinkler pipe into sterile 1-L Nalgene bottles. Post collection, soil and water samples were sent to the University of California (UC) Davis Analytical Lab for chemical analysis. Soil samples were analyzed for total organic carbon (TOC), total nitrogen (N), nitrates (NO_3_), phosphate (P), potassium (K), sodium (Na), calcium (Ca), magnesium (Mg), cation exchange capacity (CEC), organic matter (OM), pH, percentage sand, percentage silt, and percentage clay. Irrigation water and tail water samples were tested for pH, electrical conductivity (EC), sodium adsorption ratio (SAR), Ca, Mg, Na, chlorine (Cl), boron (B), bicarbonate (HCO_3_), carbonate (CO_3_), total organic carbon (TOC), total nitrogen (N), ammonium (NH_4_), nitrates (NO_3_), phosphorus (P), sulfates (SO_4_), total suspended solids (TSS), and dissolved organic carbon (DOC). Post chemical analysis, all water samples were stored at −20°C prior to use in experiments. Soil samples were stored in sealed plastic bags and held at room temperature prior to use in experiments (up to 12 months).

### 2.4 Assessment of stability of human NoV surrogates in well water, tail water, and ultrapure water

TV and MNV virus stocks were prepared to have a normalized titer of 1 × 10^6^ PFU/ml. Aliquots (50 ml) of tail water, well water, and ultrapure water were inoculated with TV or MNV to achieve a starting titer of 10^5^ PFU/ml. Samples were stored for 28 days in incubators set at three different temperatures to mimic the average (19°C), minimum (11°C), and maximum (24°C) water temperatures measured in the reservoir where tail water samples were collected. There were six replicates for TV and MNV in each type of water held at each of the three temperatures. Inoculated well, tail, and ultrapure water held at 19°C was sampled at days 0 (immediately after inoculation), 1, 2, 3, 7, 14, 21, and 28. Inoculated well, tail, and ultrapure water held at 11 and 24°C was sampled at days 0 (immediately after inoculation), 1, 2, 14, and 28. A 2-ml sample was collected at each time point from each replicate and the level of infectious virus was determined by plaque assay. To monitor the pH of each water type, uninoculated samples of water were stored at each of the three temperatures and pH was measured at days 0, 1, 2, 3, 7, 14, 21, and 28.

### 2.5 Determination of water quality impact on human NoV surrogate stability in soil

Soil collected from the central region of the Salinas Valley was sieved through 0.17-mm mesh to break up large soil particles before use. Soil (2 kg) was then added to separate 30 × 30 cm polyethylene bags. TV and MNV were inoculated into 600 ml of either tail water, well water, or ultrapure water to achieve a titer of 10^5^ PFU/ml. Treatment groups were TV + well water, TV + tail water, TV + ultrapure water, MNV + well water, MNV + tail water, and MNV + ultrapure water. Each 600-ml sample of virus-inoculated water (either well, tail, or ultrapure) was added to bagged soil (2 kg) and then mixed to achieve 100% saturation or 30% water holding capacity (WHC). Aliquots of the inoculated soil were then distributed into plastic containers (500 g of wet soil per container × 6 containers per treatment group). The target titer for each virus was 1.15 × 10^7^ PFU/container (2.3 × 10^4^ PFU/g of soil). Containers were placed on tray flats and held in an environmental chamber (PGR15, Conviron, Pembina, ND) with a light intensity of 230 μmol/m^2^·s^2^ using sun white spectrum 12W T5 LED lamps. The chamber was maintained at a constant relative humidity (60% RH) and daily temperatures of 22°C (for 12 h, with light) and 18°C (for 12 h, without light) (Moyne et al., 2013). The soil saturation (moisture) level was monitored daily with a moisture analyzer (HG63, Mettler Toledo, Columbus, OH) and by measuring the decrease in total container weight. An average soil water saturation of 62% was maintained by bottom watering with ultrapure water every 2 or 3 days. Soil was sampled to determine the level of virus infectivity on days 0 (immediately after inoculation), 1, 3, 7, 14, 21, and 28. Soil (1 g dry weight; 1.3 g saturated weight) was transferred to a sterile 15-ml conical tube and then 5 ml of sterile phosphate buffered saline (PBS) was added. Soil samples were treated for three cycles of vortexing for 1 min followed by placing on ice for 30 sec. Samples were then centrifuged at 3000*×g* for 20 min and the virus-containing supernatant was collected. The level of infectious virus recovered from the soil was determined by plaque assay.

### 2.6 Determination of water quality impact on infectivity of TV and MNV spot inoculated on romaine lettuce leaves

Seeds of romaine lettuce (*Lactuca sativa*, Parris Island Cos) were planted in plug trays and germinated in a controlled environmental chamber located in the Plant Sciences Department at the University of California Davis. Ten days after germination, plugs were transplanted into 4-inch (10.6 cm) containers containing Fafard’s 3B potting mix (Sungro, Agawam, MA). At 45 days after germination, lettuce plants were transferred to an environmental growth chamber located in a biosafety level–2 laboratory in the Food Science and Technology Department at UC Davis. The lettuce was maintained under conditions of 12 h of light and 12 h of darkness, and daily temperatures of 22°C (for 12 h, with light) and 18°C (for 12 h, without light) and a constant relative humidity of 60%. The lettuce containers were kept in tray flats, and were bottom watered every 5 days with 1,000 ml of ultrapure water.

TV and MNV (stock virus titer: 1 × 10^7^ PFU/ml) were diluted 1:10 (v/v) in either tail water, well water, or ultrapure water. Plants were divided into treatment groups TV + well water, TV + tail water, TV + ultrapure water, MNV + well water, MNV + tail water, and MNV + ultrapure water, with six replicates per treatment. Individual leaves were inoculated with 10 × 2-µl drops (2 × 10^5^ PFU/leaf). Plants inoculated with the same organism and water were grouped in one tray flat to avoid cross contamination. Inoculated leaves were sampled at days 0 (immediately after inoculation), 1, 2, 3, 4, 5, 6, and 7. For sampling, inoculated leaves were separated from the plants by hand and transferred into individual 207-ml (7-oz) Whirl-Pak filter bags with 6 ml of PBS per bag. To recover the viruses, the bags containing leaves were pummeled in a stomacher (Corning Gosselin Blender, 400 mL, 115V) for 2 min at fast speed. The virus-containing aqueous homogenates collected at days 0, 1, 2, 3, 4, 5, 6, and 7 were then transferred to separate collection tubes for determination of viral titer at different time points by plaque assays. A second experiment was conducted to assess viral infectivity over 28 days. Leaves were inoculated as described above and were sampled at days 0 (immediately after inoculation), 1, 2, 7, 10, 14, and 28.

### 2.7 Data analysis

Statistical analyses were conducted with GraphPad Prism 8 on log-transformed data for TV and MNV plaque assay counts. Effects of water type and time on the TV and MNV titers were assessed by Wilcoxon signed rank test and by analysis of variance (ANOVA), followed by a multiple comparison with a Tukey test for normally distributed data. Results with *P* values <0.05 were considered statistically significant.

## 3 Results

### 3.1 Impact of water quality on TV and MNV infectivity

The pH values of water samples measured at day 0 were 7.36 ± 0.78 for ultrapure water (not shown), 7.63 ± 0.15 for well water, and 7.76 ± 0.16 for tail water (**Table 1**). The tail water used in the study had turbidity of 41 ± 0.00 mg/L of total suspended solids (TSS) (**Table 1**). Compared with tail water, well water had lower concentrations of dissolved organic carbon (DOC), total organic carbon (TOC), and TSS but higher total nitrogen (**Table 1**).

**Table 1:** Water quality chemical analysis for tail water and well water collected from a single farm in the Salinas Valley.

Characteristic	Tail water*^a^*	Well water*^a^*
pH	7.76 ± 0.16	7.63 ± 0.15
EC (μS/cm)	1.07 ± 0.00	0.90 ± 0.01
SAR	1.60 ± 0.00	0.80 ± 0.00
Ca^2+^ (mEq/L)	3.99 ± 0.05	5.32 ± 0.05
Mg^2+^ (mEq/L)	3.39 ± 0.04	2.42 ± 0.01
Na^+^ (mEq/L)	3.13 ± 0.04	1.62 ± 0.02
Cl^-^ (mEq/L)	1.98 ± 0.01	1.45 ± 0.04
B^3+^ (mEq/L)	0.06 ± 0.00	0.04 ± 0.00
HCO_3_^-^ (mEq/L)	1.60 ± 0.80	3.00 ± 0.00
CO_3_^2-^ (mEq/L)	0.20 ± 0.00	<0.10 ± 0.00
TOC (mg/L)	15.75 ± 0.21	2.15 ± 0.07
Total N (mg/L)	5.02 ± 0.01	20.80 ± 0.11
NH_4_^+^ (mg/L)	0.73 ± 0.01	<0.05 ± 0.00
NO_3_^-^ (mg/L)	2.53 ± 0.00	22.14 ± 0.06
PO_4_^3-^ (mg/L)	<0.05 ± 0.00	<0.05 ± 0.00
Total P (mg/L)	0.20 ± 0.01	<0.10 ± 0.00
SO_4_^2-^ (mg/L)	118.75 ± 0.21	51.75 ± 0.35
TSS (mg/L)	41.00 ± 0.00	14.00 ± 0.00
DOC (mg/L)	12.05 ± 0.07	1.90 ± 0.28

^a^ Values are means ± standard deviation (n = 2).

EC, electrical conductivity; SAR, sodium absorption ratio; TOC, total organic carbon; TSS, total suspended solids; DOC, dissolved organic carbon.

**Figure 1:**
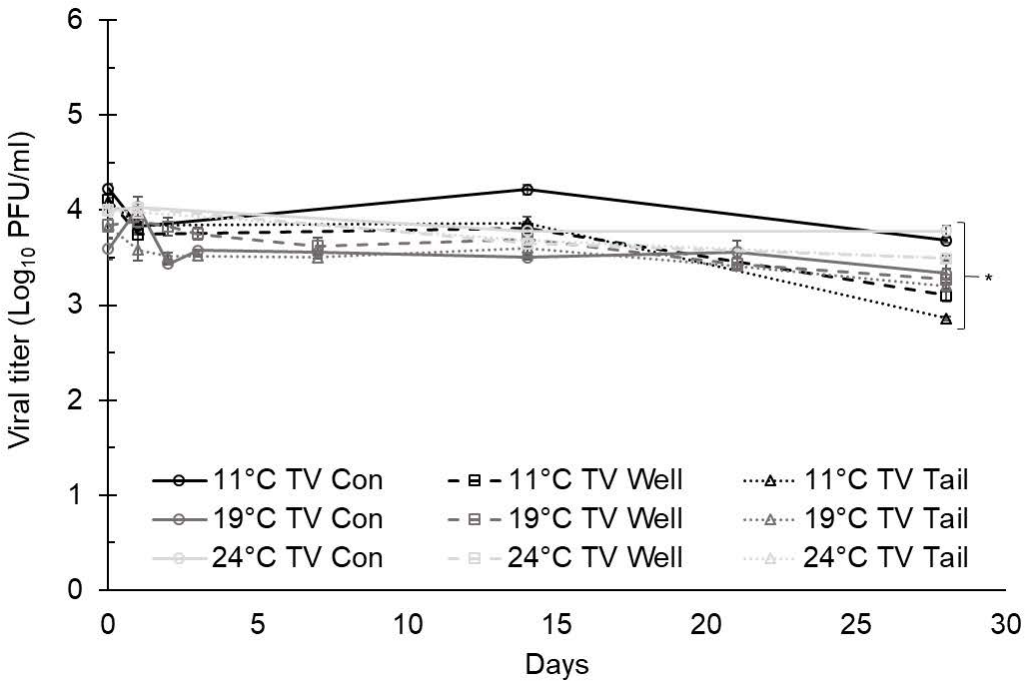
Tulane virus (TV) infectivity in ultrapure (control), well, and tail water samples held at 11, 19, and 24°C and assessed for 28 days. Viral titer is reported as PFU/ml. Data points are the average of six replicates. Error bars represent ± standard deviation. * denotes significant difference (p<0.05) compared to day 0 within the same treatment group.

**Figure 2:**
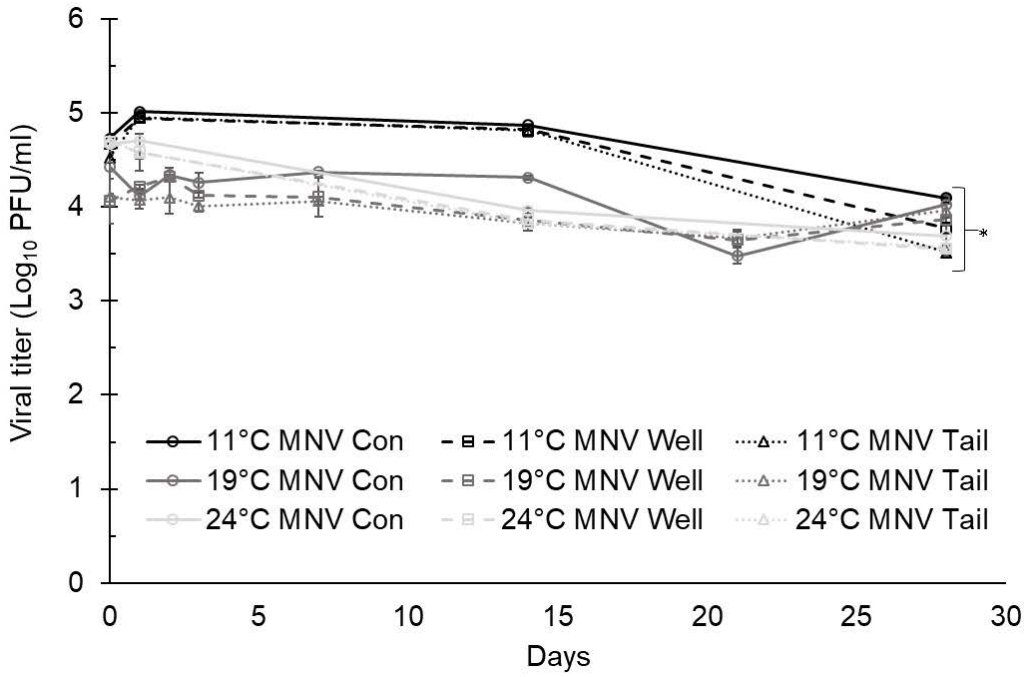
Murine norovirus (MNV) infectivity in ultrapure (control), well, and tail water samples held at 11, 19, and 24°C and assessed for 28 days. Viral titer is reported as PFU/ml. Data points are the average of six replicates. Error bars represent ± standard deviation. * denotes significant difference (p<0.05) compared to day 0 within the same treatment group.

At 11°C, the titers of TV at day 0 were 4.23 ± 0.05 log PFU/ml in ultrapure water, 4.11 ± 0.06 log PFU/ml in well water, and 4.09 ± 0.04 log PFU/ml in tail water (**Figure 1**). The titers of MNV at day 0 were 4.72 ± 0.05 log PFU/ml in ultrapure water, 4.67 ± 0.05 log PFU/ml in well water, and 4.52 ± 0.06 log PFU/ml in tail water (**Figure 2**). A maximum 1.5-log reduction was observed for TV and MNV over 28 days (compared with day 0) when stored at 11°C (**Figures 1**, **2**).

At 19°C, the titers of TV at day 0 were 3.59 ± 0.11 log PFU/ml in ultrapure water (control), 3.84 ± 0.07 log PFU/ml in well water, and 3.82 ± 0.12 log PFU/ml in tail water (**Figure 1**). The titers of MNV at day 0 were 4.43 ± 0.13 log PFU/ml in ultrapure water, 4.96 ± 0.24 log PFU/ml in well water, and 4.10 ± 0.04 log PFU/ml in tail water (**Figure 2**). After 28 days at 19°C, reductions of less than 1 log were observed for both TV and MNV (compared with day 0) in all three water types (**Figures 1**, **2**).

At 24°C, the titers of TV at day 0 were 3.99 ± 0.00 log PFU/ml in ultrapure water, 4.00 ± 0.02 log PFU/ml in well water, and 3.99 ± 0.04 log PFU/ml in tail water (**Figure 1**). The titers of MNV at day 0 were 4.67 ± 0.04 log PFU/ml in ultrapure water, 4.69 ± 0.04 log PFU/ml in well water, and 4.71 ± 0.07 log PFU/ml in tail water (**Figure 2**). A maximum 1.5-log reduction was observed for TV and MNV over 28 days (compared with day 0) when stored at 24°C (**Figures 1**, **2**).

Statistically significant differences in the virus titer were observed for both TV and MNV at day 0 compared with day 28 in all water types at each temperature evaluated (**Figures 1**, **2**). Statistical analysis using Tukey’s multiple comparisons found no significant differences among viral reductions based on the type of water or holding temperature (data not shown).

### 3.2 Influence of water quality on the infectivity of TV and MNV in soil

**Figure 3:**
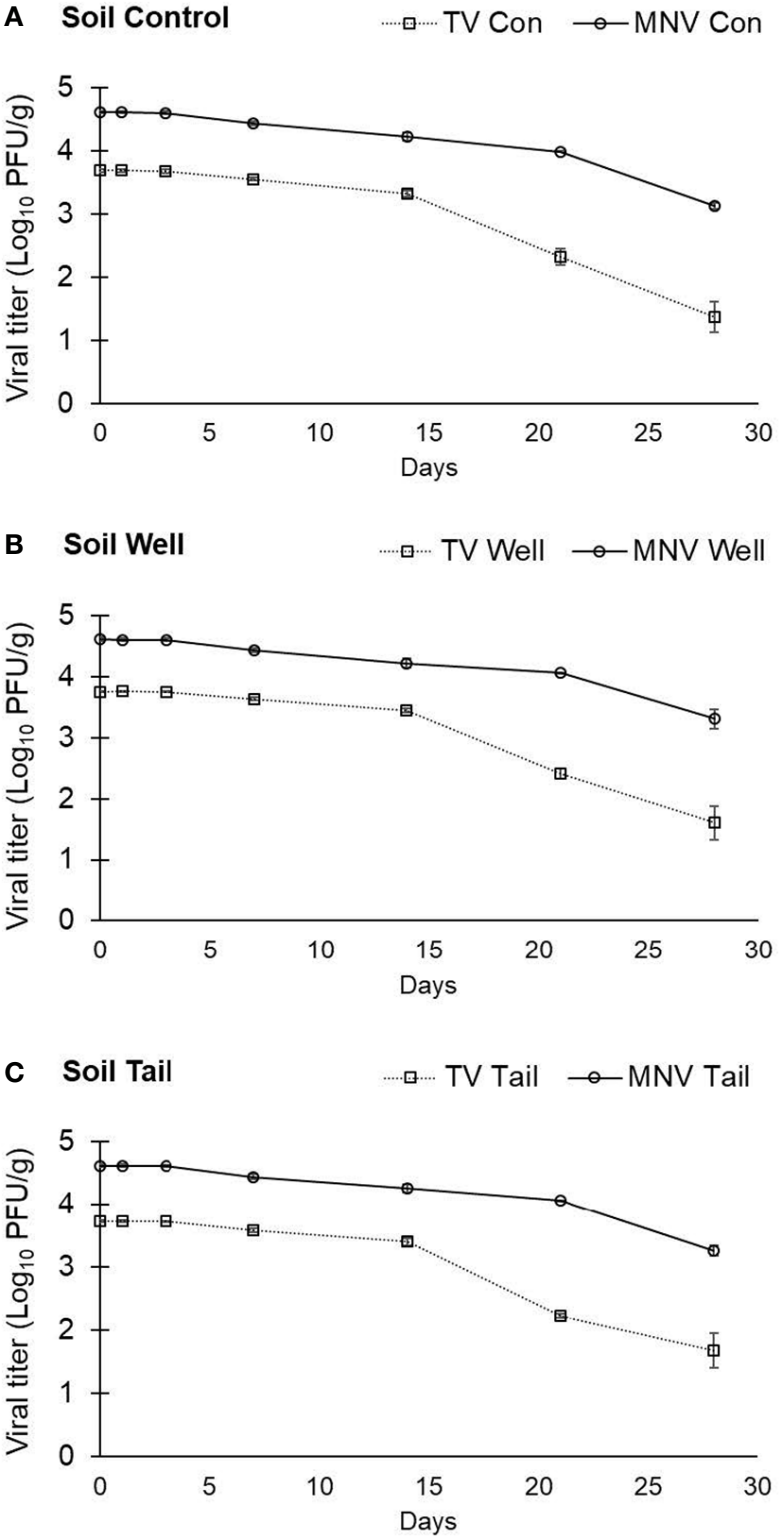
Infectivity of TV and MNV in sandy loam soil over a 28-day period. TV or MNV were suspended in **(A)** ultrapure water (control), **(B)** well water, and **(C)** tail water and the suspensions were separately mixed uniformly into soil. Viral titer is reported as PFU/g. Data points are the average of six replicates. Error bars represent ± standard deviation.

Based on soil composition analysis (**Table 2**), the soil type collected from a Salinas Valley farm was determined to be Arroyo Seco gravelly loam. The titer of TV detected at day 0 in soil was determined to be 3.69 ± 0.02 log PFU/g using ultrapure water as the suspension medium, 3.75 ± 0.01 log PFU/g using well water as the suspension medium, and 3.73 ± 0.02 log PFU/g using tail water as the suspension medium (**Figure 3**). Recovery of TV applied to the soil declined over 28 days, with a 2- to 2.5-log reduction compared with day 0, depending on the suspension medium (water type) used (**Figure 3**), but reductions of TV in soil at any time point were not significantly different.

**Table 2:** Chemical characteristics of soil samples collected (from one farm) in the Salinas Valley.

Characteristic	Soil*^a^*
pH	7.85 ± 0.01
TOC (%)	0.97 ± 0.00
Total N (%)	0.10 ± 0.00
NO_3_-N (ppm)	29.90 ± 0.00
Olsen-P (ppm)	44.90 ± 0.42
X-K (ppm)	332 ± 2.83
X-K (mEq/100 g)	0.85 ± 0.01
X-Na (ppm)	97.00 ± 0.00
X-Na (mEq/100 g)	0.42 ± 0.00
X-Ca (mEq/100 g)	16.90 ± 0.14
X-Mg (mEq/100 g)	2.98 ± 0.02
OM (LOI) (%)	1.84 ± 0.00
Sand (%)	54.00 ± 0.00
Silt (%)	26.50 ± 0.71
Clay (%)	19.50 ± 0.71

^a^ Values are means ± standard deviation (n = 2).

TOC, total organic carbon; Olsen-P, extractable phosphorus - Olsen method; X-K, exchangeable potassium; X-Na, exchangeable sodium; X-Ca, exchangeable calcium; X-Mg, exchangeable magnesium; OM (LOI), organic matter – Loss-On-Ignition method.

The titer of MNV detected at day 0 in soil was 4.62 ± 0.02 log PFU/g with ultrapure water, 4.62 ± 0.03 log PFU/g with well water, and 4.62 ± 0.02 log PFU/g with tail water as the suspension medium (**Figure 3**). Recovery of MNV from soil declined over 28 days, with a less than 1.5-log reduction compared with day 0 regardless of water type (**Figure 3**). No significant difference in the stability of MNV in soil was observed when comparing different suspension mediums (water types). However, there was a significant difference in the stability of the two viruses in soil, with significantly higher log reductions at both day 21 and day 28 for TV than for MNV in all water types (data not shown).

### 3.3 Recovery of infectious virus from romaine lettuce leaves

**Figure 4:**
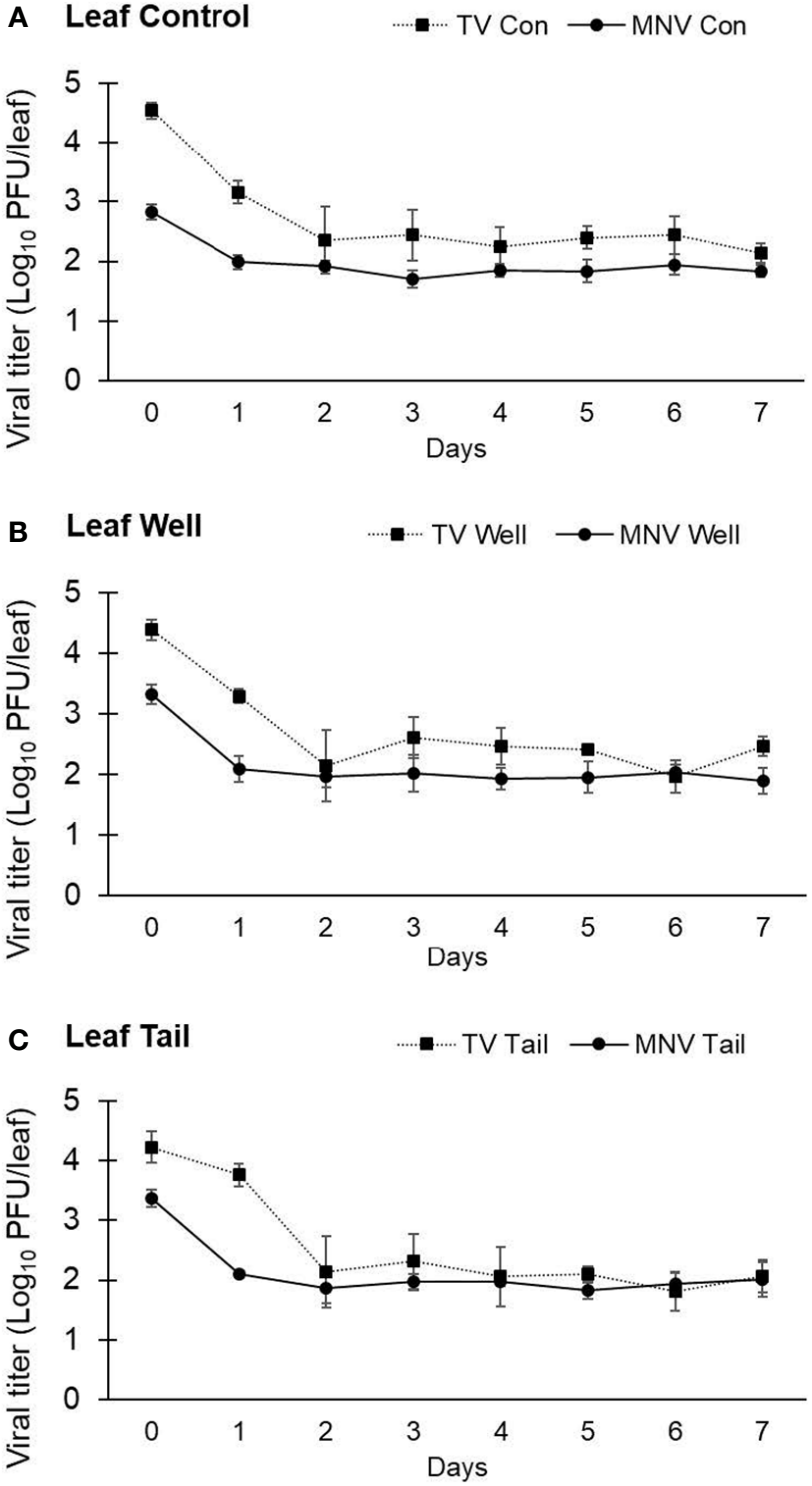
Impact of water quality on the recovery of infectious human norovirus surrogates from the surface of romaine lettuce leaves. Infectivity of TV and MNV on the surface of lettuce leaves was assessed for 7 days (trial 1) when suspended in **(A)** ultrapure water, **(B)** well water, and **(C)** tail water. Viral titer is reported as PFU/leaf. Data points are the average of six replicates. Error bars represent ±standard deviation.

In the first trial lasting 7 days, the titer of TV recovered from lettuce leaves at day 0 was determined to be 4.54 ± 0.13 log PFU/leaf using ultrapure water, 4.38 ± 0.16 log PFU/leaf using well water, and 4.23 ± 0.26 log PFU/leaf using tail water as the suspension medium (**Figure 4**). The level of infectious TV recovered from lettuce leaves declined by approximately 2 log at day 2 post inoculation and remained stable at this level for the duration of study period. The level of TV recovered from lettuce leaves at day 7 was 2.14 ± 0.16 log PFU/leaf using ultrapure water, 2.46 ± 0.16 log PFU/leaf using well water, and 2.06 ± 0.28 log PFU/leaf using tail water as the suspension medium. There was no significant difference in the level of TV recovered from lettuce leaves using any of the three water types as the suspension medium.

The titer of MNV detected at day 0 on lettuce leaves was 2.83 ± 0.12 log PFU/leaf with ultrapure water, 3.33 ± 0.16 log PFU/leaf with well water, and 3.37 ± 0.14 log PFU/leaf with tail water as the suspension medium (**Figure 4**). MNV recovered from lettuce leaves declined by approximately 1 log at day 1 post inoculation and remained stable at this level for the duration of study period. The level of MNV recovered from lettuce leaves at day 7 was 1.84 ± 0.10 log PFU/leaf using ultrapure water, 1.89 ± 0.22 log PFU/leaf using well water, and 2.01 ± 0.29 log PFU/leaf using tail water as the suspension medium (**Figure 4**). As observed with TV, there was no significant difference in the level of MNV recovered from lettuce leaves using any of the three water types as the suspension medium. The recovery of TV from lettuce leaves on day 0 was approximately 1 log higher than the level of MNV recovered from lettuce leaves at day 0. Using the titer of virus at day 0 to calculate the log reduction at each time point resulted in a significantly higher log reduction in TV compared with MNV at each time point. However, both viruses were recovered at a level of approximately 2 log PFU/leaf at day 7.

**Figure 5:**
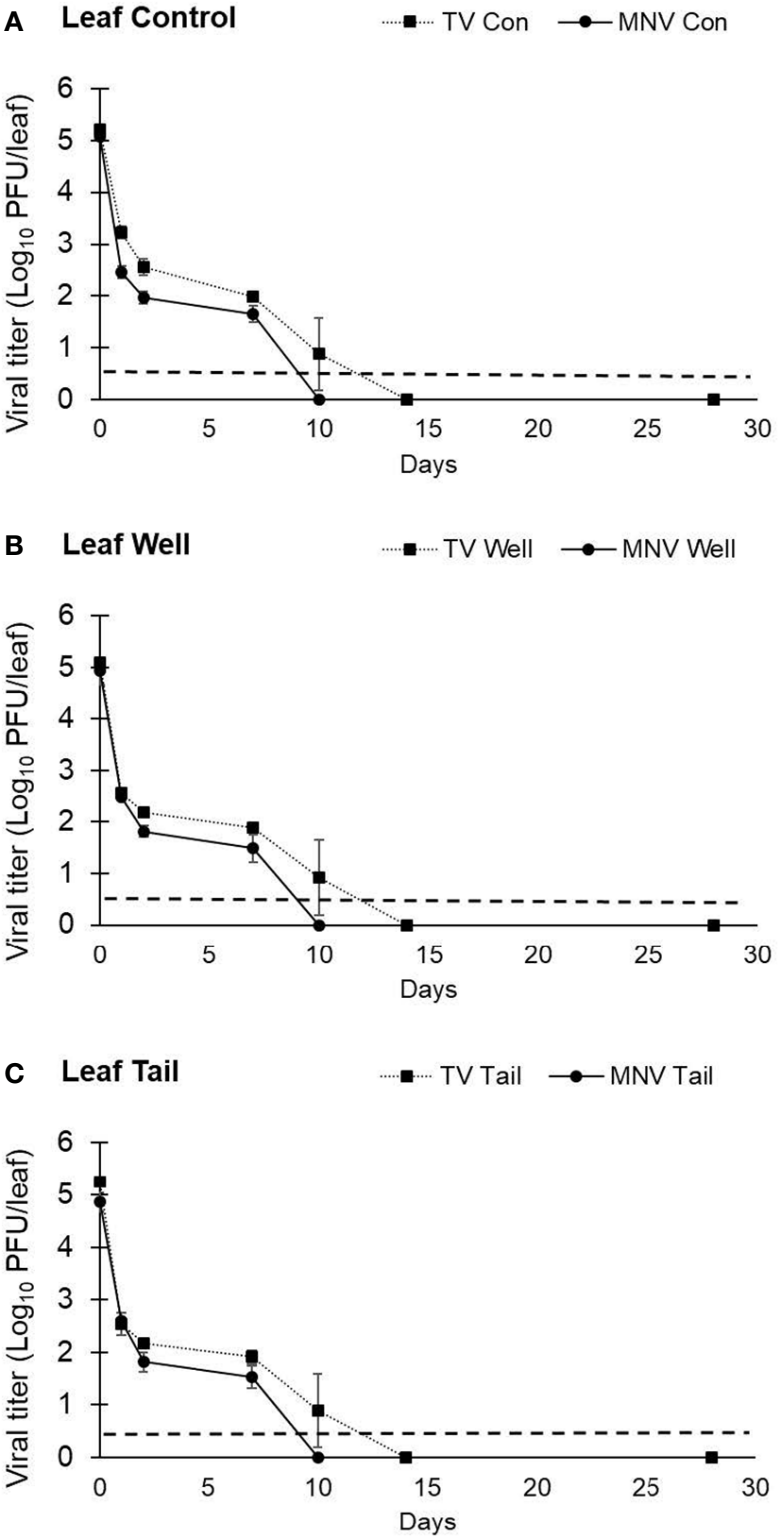
Impact of water quality on the recovery of infectious human norovirus surrogates from the surface of romaine lettuce leaves. Infectivity of TV and MNV on the surface of lettuce leaves was assessed for 28 days (trial 2) when suspended in **(A)** ultrapure water, **(B)** well water, and **(C)** tail water. Viral titer is reported as PFU/leaf. Data points are the average of six replicates. Error bars represent ± standard deviation.

A second trial with lettuce was set up to assess TV and MNV viral infectivity beyond 7 days. The same inoculation procedure used in trial 1 was used in trial 2, but infectivity was assessed for 28 days. The titer of TV recovered from lettuce leaves at day 0 was determined to be 5.20 ± 0.06 log PFU/leaf using ultrapure water, 5.09 ± 0.09 log PFU/leaf using well water, and 5.25 ± 0.09 log PFU/leaf using tail water as the suspension medium (**Figure 5**). The titer of MNV detected at day 0 on lettuce leaves was 5.08 ± 0.08 log PFU/leaf with ultrapure water, 4.93 ± 0.27 log PFU/leaf with well water, and 4.86 ± 0.18 log PFU/leaf with tail water as the suspension medium (**Figure 5**). Plaque assays detected no viable TV at day 14 and no viable MNV at day 10. The initial titer of each virus in trial 2 was significantly higher compared with trial 1, though the targeted initial concentration was the same. In trial 2, at each time point where infectious virus was recovered there was no significant difference in log reduction between MNV and TV.

## 4 Discussion

As obligate intracellular parasites, viruses do not increase in numbers outside of a permissive host cell. In the environment, including water, soils, and surfaces, virus levels only remain static or decline. As such, environmental factors, including temperature, humidity, and UV exposure, play an important role in dictating the infectivity of viruses outside of the host (Pinon and Vialette, 2018). Moreover, biochemical interactions of the virus particle with substrates in the environment can influence their mobility as well as potentially lead to viral inactivation. In our study, there were slight increases in viral titer observed at some timepoints, however this non-significant increase is related to variability inherent to the viral recovery process.

Temperature is recognized as a key variable influencing virus environmental stability (Akin et al., 1975; Lo et al., 1976; Yates et al., 1985). Generally, the stability of non-enveloped viruses increases at temperatures below normal body temperature (37°C). At temperatures close to freezing, non-enveloped viruses have been shown to retain infectivity for months to years (Biziagos et al., 1988; Dublineau et al., 2011). In this study, there was no significant difference in the inactivation kinetics of TV and MNV in water when stored at 11, 19, and 24°C. Populations of both TV and MNV were stable in water over 28 days. These results correlate well with previous studies using human NoV clinical isolates. Human NoV GII.1 RNA levels were detectable for up to 20 weeks in wastewater and stable for up to 1 year in drinking water at temperatures of 3 and 21°C, respectively (Kauppinen and Miettinen, 2017). Human NoV GI.1 suspended in water stored at room temperature (18°C) for 61 days remained infective in human volunteers (Seitz et al., 2011).

The pH of a solution plays an important role in dictating the biochemical interactions of the viral capsid protein with molecules and ions in the environment. The protonation of the capsid proteins suspended in water leads to the formation of electrically charged surfaces (Gerba, 1984; Brown et al., 1999; Al-Abadleh and Grassian, 2003; Michen and Graule, 2010). The surface charge is pH dependent. For viruses, the pH at which the net surface charge is neutral is called the isoelectric point (Parks, 1965; Gerba, 1984; Brown et al., 1999; Al-Abadleh and Grassian, 2003; Michen and Graule, 2010). Below the isoelectric point, the viral capsid protein has an overall positive charge and above the isoelectric point, the viral capsid has an overall negative charge. The isoelectric point of the human NoV has been shown to be between pH 5.5 and 6, with variation dependent on virus strain (Goodridge et al., 2004). Therefore, depending on the pH of agricultural water (pre- or postharvest) different foodborne pathogenic viruses may have vastly differing surface changes, which can influence their sorption and desorption profiles in liquid and on solid surfaces (Sobsey et al., 1980). The water samples used in this study all had pH values above 7, indicating that viral particles likely carried a negative surface charge across all experiments.

Ultrapure water is 18.2 MΩ.cm (no other ions present other than those created by the dissociation of water) and has a TOC value below 5 ppb. Unsurprisingly, well water and tail water had significantly higher values of water quality parameters compared with ultrapure water. Tail water had significantly higher values for DOC, TOC, and TSS compared with well water. Total N was higher for well water compared with tail water. However, none of these differences in water quality parameters played a significant role in recovery or infectivity of TV and MNV in the water samples at the temperature ranges evaluated in this study.

Infectious TV and MNV were recovered from soil samples at 28 days post inoculation. The level of TV declined by approximately 2 logs and MNV by approximately1 log over 28 days compared with virus levels recovered on day 0 immediately after inoculation. Several factors can influence virus recovery and infectivity in soil, including soil type, water saturation, pH, and organic matter. Fine-textured soils have been reported to adsorb viruses more readily than coarse-textured soils (Sánchez and Bosch, 2016). Fine-textured soils are also known to have more cation exchange capacity and enhance the adsorption of organic matter (Hatten and Liles, 2019). The soil collected from the Salinas Valley was characterized as sandy loam, which consisted of 54% sand, 26% silt, and 20% clay. Sandy loam is considered a coarse-textured soil (van Es et al., 2017). The decline in virus titer observed on day 21 and day 28 compared with other sampling time points may be related to loss of virus particles from the coarse-textured soil matrix, rather than inactivation of viral particles.

The soil was maintained at an average soil water saturation of 62% throughout the study period. A highly water saturated soil also allows for more viral movement, as all the pores in the soil are open and the virus has less interaction with the soil particles (Bagdasaryan, 1964; Santamaría and Toranzos, 2003). The coarse soil, combined with a high level of soil saturation, may have contributed to reductions in viral recovery over the duration of the study period. Another factor that may impact viral recovery from soils is the pH of the soil/water matrix. The soil used in this study had a pH of 7.85. This finding, coupled with the pH of the water suspension matrix, suggests that the viral capsid will have a negative surface charge. Therefore, the virus may adhere to positively charged materials found in the soil and be tightly bound in the soil matrix (Sobsey et al., 1980). This interaction between the virus capsid and positively charged components of the soil may lead to reduced recovery of viruses from soil, due to adhesion of virus to the soil rather than to inactivation of the virus.

Inactivation of viruses in soil or on leaf surfaces in also a possibility. Soil samples and lettuce plants were maintained in a growth chamber mimicking daily light and temperature cycles observed in the Salinas Valley. The temperature fluctuated between 11 and 24°C. The results from the water analysis indicate viruses will be stable within this temperature range; however, cycling of temperature was not a variable examined in the water experiments. Additionally, inoculated soils were subjected to 12-h sun white spectrum LED light cycling at an intensity of 230 μmol/m^2^·s^2^ (Moyne et al., 2013). White light has a spectrum of approximately 390 to 700 nm. Viruses can be inactivated by ultraviolet (UV)-C (100 to 280 nm) and far UV-C (222 nm) wavelengths of light, however these wavelengths are not included in LED lights utilized in growth chambers. While further investigation is warranted, exposure to visible light may have played a role in the viral titer reductions in soil and on leaf surfaces.

Leafy greens are considered a high-risk food for human NoV contamination (Marsh et al., 2018). Previous studies have shown that viruses can survive for extended periods on the surface of leafy greens. Porcine sapovirus has been shown to remain infectious on leafy green surfaces during postharvest storage at 4°C with a 3.7-log reduction after storage for 7 days (Wang et al., 2012). MNV inoculated onto lettuce surfaces showed a 3-log reduction in infectivity over 14 days when stored at room temperature (Escudero et al., 2012). On preharvest spinach, TV and MNV were found to remain infectious for up to 7 days (Hirneisen and Kniel, 2013b). MNV infectivity on stainless steel coupons was found to be reduced by 2.28 log_10_ PFU/coupon over 28 days at room temperature, however residual infectious virus was recovered from the coupons at the 28 day timepoint (Kim et al., 2014). In other works, MNV stability was evaluated on three different surfaces (stainless steel, ceramic, and formica) and iceberg lettuce leaves in the same study. Infectivity was found to have decreased by 3 logs within 21 days on food contact surfaces and by 3.0 logs on the surface of lettuce leaves in 14 days at room temperature, indicating enhanced infectivity or recovery from food contact surfaces compared to lettuce (Escudero et al., 2012).

Viruses have been shown to adsorb to produce surfaces and be resistant to removal (Gandhi et al., 2010; Esseili et al., 2012; DiCaprio et al., 2015b). The surfaces of lettuce leaves have complex micro-scale topography and are covered by a cuticle that has a high lipid composition, resulting in a hydrophobic surface (Yeats and Rose, 2013). The hydrophobicity of a surface has been shown to play a critical role in the adsorption of viruses, due to amino acid charges or hydrophobic residues in the capsid protein (Shields and Farrah, 2002). In this study, TV and MNV declined by 2 and 1 log in the first 2 days on the surfaces of romaine lettuce leaves; no further declines were observed over the 1-week trial period. When the trial was extended to 4 weeks, TV and MNV declined by ≥2 log at day 1, and were below the limit of detection (total ~4.5 log reduction) by day 10 (MNV) or day 14 (TV). The lettuce leaf surface topographic complexity is known to increase with time, as is the leaf surface coverage with the hydrophobic cuticle (Doan et al., 2020; Kane et al., 2020). Therefore, the lack of detection of infectious virus with increasing leaf age may be due to lack of recovery from the leaf surface rather than inactivation. Extending the study period also increases the exposure to white light, fluctuating temperatures, and may enhance potential for desiccation. While these factors independently do not decrease human NoV infectivity, the exposure to all variables in conjunction may have led to inactivation on the virus on the surface of lettuce leaves.

The virus structure can play a role in its interaction with environmental surfaces. Two human NoV surrogates were compared in the current study, and both exhibited similar stability in a water matrix over 28 days. While tail water was utilized in this study as a representative lower quality water source, only one type of tail water was used in experiments, which may limit conclusions related viral stability in different sources of water. In soil systems, the level of recoverable TV declined by approximately 2 logs compared with an approximate 1-log reduction in MNV titer under the same conditions over 28 days. This could be due differences in relative stability in the environment between TV and MNV. Previous work comparing the stability of these two surrogates in dry standard potting mix, showed that TV was reduced by 1 log over 14 days compared to 2 logs for MNV under the same conditions, an inverse of the results of the current study (DiCaprio et al., 2015). Differences observed in this study therefore may also be due soil saturation levels or soil type itself leading to the reduction in viral recovery rates. TV may be more susceptible to adsorbing components of the soil matrix compared with MNV, and may account for the enhanced reduction of titer observed on days 21 and 28 for TV compared with MNV. Though both viruses are similar in size and buoyant density, they differ in the attachment molecules recognized to initiate viral infection of a host cell: TV recognizes HBGAs, while MNV recognizes sialic acids (Hirneisen and Kniel, 2013a). There is potential that moieties similar to HBGAs are present in soil and may have led to TV specific adhesion to the soil matrix. The isoelectric points for MNV and TV are not well defined and it is possible that differences in the isoelectric point may have played a role in the differences observed. Only one soil type was included in the study and therefore this effect may not be observed when using soil samples from other locations.

While the majority of foodborne viruses, including human NoV, are not known to be zoonotic, the viruses can impact surface water sources through improperly maintained septic systems, inadequate water treatments, or other mechanisms where human waste can enter the water system. At least one foodborne virus, hepatitis E virus (HEV), is zoonotic and wildlife is known to be a reservoir (ex: feral swine, deer, rabbits) for HEV and often impact the microbial quality of surface water (Harrison and DiCaprio, 2018). Using tail water collected from a production farm provides information related to the relative stability of enteric viruses in low quality surface water compared to traditional water sources used in production systems and can assist in future development of guidance for utilizing alternative water sources. Overall, human NoV surrogates are highly stable in potential irrigation water sources at temperatures relevant to the leafy green production. The surrogates remain infectious in soil for up to 28 days when delivered in irrigation water. Moreover, infectious human NoV surrogates are recovered from romaine lettuce for extended periods (10 days for TV; 7 days for MNV) when suspended in irrigation water and applied to leaf surfaces. Irrigation water chemical quality did not have a significant impact on human NoV surrogate stability in the present study. This information may help to inform best practices when conducting site assessments for irrigation water sources to mitigate risk associated with foodborne viruses. These data provide insight into remediation strategies for fields impacted by human sewage infiltration to establish appropriate die-off intervals for foodborne viruses.

## Data availability statement

The raw data supporting the conclusions of this article will be made available by the authors, without undue reservation.

## Author contributions

ED, LH, and AM contributed to the study conception and design. Funding was acquired by LH. Material preparation, data collection and analysis were performed by XW and TR. The first draft of the manuscript was written by XW, TR, and ED and all authors commented on previous versions of the manuscript. All authors contributed to the article and approved the submitted version.
